# Validation of a modified questionnaire to assess Ukrainian Family Physicians’ readiness to implement the evidence-based screening recommendations into their clinical practice, using a mixed method study

**DOI:** 10.1186/s12875-022-01818-4

**Published:** 2022-09-07

**Authors:** I. Shushman, A. Kolesnyk, P. Kolesnyk, G. Kuodza, I. Mykyta, S. Bayen, T. Frese

**Affiliations:** 1 Department of Family Medicine and Outpatient Care, Medical Faculty #2, Education Scientific Family Medicine Training Center, Uzhhorod National University, Uzhgorod, Ukraine; 2 Department of General Practice, University of Lille, UFR3S Lille, France; 3 Department of General Practice & Family Medicine, Medical Faculty, Martin-Luther University, Magdeburger Straße 8, 06112 Halle /Saale, Germany

**Keywords:** Validation, Questionnaire, Family medicine, Evidence-based screening, Guidelines

## Abstract

**Background:**

Ukrainian Family Doctors' knowledge and readiness for the implementation of new guidelines recommendations into practice has to be evaluated and taken into consideration, meanwhile they often use the old protocols of annual checkups. This study aimed to perform a linguistic and cultural validation of Ukrainian adopted questionnaire designed on the German prototype “Readiness of general practitioners to recommend and implement evidence-based screening recommendations questionnaire”.

**Methods:**

This was a mixed method study. The English version of the original German prototype questionnaire was translated into Ukrainian with forward–backward method. Additionally Ukrainian version was modified by including an extra questions about evidence based screening of cardiovascular risks, infectious diseases, type 2 diabetes mellitus, depression, and some old-fashioned annual checkups which are still commonly used in routine family doctors’ practice. During the face validation process, 10 practicing general practitioners assessed all the items in the questionnaire as adequately structured, grammatically correct, and understandable. During qualitative part of content validation process 11 experts found and corrected some grammatical errors, indicated that items of the questionnaire were comprehensible and related to Ukrainian culture. During the quantitative part of content validation process experts accessed 5 of 6 items as essential, relevant, and clear. Internal consistency of the items, assessed by using Cronbach’s alpha method was acceptable. To access how stable can be results provided by the questionnaire, test–retest reliability was performed, where 19 general practitioners filled in the same questionnaire twice over a period of two weeks.

**Results:**

In our study CVR > 0.79 and CVI > 0.59 were acceptable. The internal consistency was evaluated by using Cronbach’s alpha method and had to be above 0.7. According to the test–retest reliability results of weighted kappa and Cohen's kappa coefficients, test–retest agreement of the questionnaire was moderate for 29%, substantial for 38%, and almost perfect for 5% of the items (*p* ≤ 0.05). Kappa coefficients were not computed for 10% of items as there was no variability in the assessments.

**Conclusions:**

The Ukrainian version of the questionnaire can be used for the assessment Ukrainian Family Doctor’s readiness to implement the evidence-based screening recommendations into their clinical practice.

**Supplementary Information:**

The online version contains supplementary material available at 10.1186/s12875-022-01818-4.

## Background

In a systematic analysis for the Global Burden of Disease Study 2019 it is highlighted that the global burden of chronic non-communicable and communicable diseases is increasing [1]. Cardiovascular and oncological diseases keep leading positions in the list of the most common causes of death in Europe [2, 3]. Recent years many European countries increased their focus on preventive measures and a growing trend towards more screening for non-communicable diseases is there [4]. Four separate categories of prevention have been defined by the World Organization of National Colleges and Academies of Family Medicine (WONCA) and the International Dictionary for General/Family Practice in 2003 (See Table 1) [5, 6].

**Table 1:** Categories of prevention defined by WONCA

**Primary prevention**-an action aimed to avoid an occurrence of some diseases (e.g.vaccination)	**Secondary prevention-** an action aimed to detect a disease at an early stage in absolutely asymptomatic person (e.g. screening)
**Tertiary prevention**- an action aimed to prevent an occurrence of different complications of already diagnosed disease (e.g. prevention of complications of arterial hypertension)	**Quaternary prevention**—an action aimed to protect patients from over-medicalization and unnecessary medical invasions

Among European countries different screening recommendations and guidelines concerning breast cancer, cervical cancer and colorectal cancer screening exist. But not all countries follow the basic criteria for screening [7].

In central and western Europe there are different governing bodies that aid, support and provide screening recommendations to the citizens. However, the situation is different in Ukraine. Previous post-Soviet non-evidence-based screening protocol was canceled by the Ukrainian Ministry of Health Care legislation in 2018 [8]. But the new one has not been offered yet [9]. The government is still searching for means of implementing and introducing various screening recommendations [9]. Clinicians' knowledge and readiness for the implementation of new guidelines recommendations into practice also have to be evaluated and taken into consideration, meanwhile they often use the old protocols of annual checkups in their routine practice [10]. Besides just providing recommendations for Family Doctors (FDs) to use, screening needs to be state-based and covered by insurance as in other European countries.

Nowadays the Ukrainian family doctors' knowledge about evidence based screening and their readiness to implement these modern screening recommendations into clinical practice is unclear.

In this aim, we needed and elaborated a questionnaire to assess Ukrainian General practitioners’ (GPs) readiness to recommend and provide modern screening strategies based on evidence instead of the old ways of post-Soviet screening. This article will describe the elaboration and validation procedure of the questionnaire about readiness to recommend and implement such screening in Ukrainian FD’ practice (See Supplementary info file).

### Objective

This study aimed to perform a linguistic and cultural validation of Ukrainian adopted questionnaire designed on the German prototype “Readiness of GPs to recommend and implement evidence-based screening recommendations questionnaire”.

## Materials and methods

This mixed method study was conducted by the research team of Family Medicine and Outpatient Care Department of Medical Faculty 2 of Uzhhorod National University, Uzhhorod, Ukraine.

### Instrument

The questionnaire that we used for our study was a modified version of the questionnaire designed by the German scientists in their research for cancer-based screening [10]. Prior German questionnaire was developed and validated in Uzhhorod, Ukraine. The original version of it included 5 questions. The permission to use the original version of the questionnaire for Ukrainian research objectives was granted by the German authors. Except oncological screening we were interested in a screening of cardiovascular diseases, type 2 diabetes mellitus, some common infectious diseases (hepatitis B and C, HIV, and others), i.e. we included extra questions using the same design and formulation as in the German prototype questionnaire. As far as the state-based screening program has not yet been implemented in Ukraine, some GPs still follow post-Soviet protocol and recommend adults to follow some routine non-evidence-based investigations during annual check-ups (for example, CBC, urine analysis, lungs X-ray) [9]. To achieve our respondents' knowledge and readiness to follow the new evidence based screening strategies comparing with the old ones, commonly used in family medicine practice as routine annual checkups, we also included the list of such investigation into the questionnaire as well.

### Translation

Two bilingual experts cooperated with the researcher to translate the questionnaire, using a forward–backward method. Firstly, two native Ukrainian experts (who had an advanced level in English) translated the original English version of the questionnaire into Ukrainian. Then, the two native English-speaking experts (who had an advanced level in Ukrainian) translated it back into English. After that, both versions were discussed and compared in an expert panel meeting and presented the final version of the Ukrainian questionnaire [11–13].

### Face validity

The adopted questionnaire was given to 10 practicing GPs with more than 5-years of practical experience. GPs had to assess if items in the questionnaire had been adequately structured if the grammar was correct if the questions were understandable. For the evaluation of the importance of questions, they were provided with a 5-point Likert scale for each of the items to range from 1 (not important at all) to 5 (highly important). Once all the results were collected, the impact score was calculated using the formula below and scores > 1.5 were considered acceptable.[14]

$$\mathrm{Impactscore}\:=\:\mathrm{Frequency}\;(\%)\:\times\:\mathrm{Importance}.$$

### Content validity

Both qualitative and quantitative phases were assessed by experts who were familiar with both the topic and the purpose of the questionnaire. We recruited 11 experts who were practicing as GPs and had experience in teaching family medicine. The experts had to assess if there were any grammatical errors, if the information was comprehensible, if each item was related to Ukrainian cultural issues and if there were any variants to improve the suggested instrument. Once all the modifications have been made, the questionnaire was given back to the experts to assess each item of the questionnaire for content validity ratio (CVR). They had to review the items with respect to being essential, useful, or unnecessary. For that purpose, experts were provided with a 4-point Likert scale ranging from 1 to 4 to assess each question for clarity and relativity. Once this was done content validity indexes (CVI) were calculated for each question.

The CVR was calculated by the ratio of the total number of experts divided by the number of experts saying item essential/total number of experts on the panel.

The CVI of each item, both for relevance and for clarity, was calculated by the ratio of the number of responses “3” or “4” in relation to the total number of responses to the item [15].

In our study CRV > 0.79 and CVI > 0.59 were acceptable.

#### Reliability

For the assessment of reliability, both the internal consistency and the test–retest reliability were evaluated. The sample size for internal consistency testing included 57 responses and was evaluated by using Cronbach’s alpha method. To prove it the internal consistency had to be above 0.7 [16].

To assess test–retest reliability the questionnaire was administered twice among 19 doctors over a period of two weeks.

Test–retest reliability was analyzed by weighted kappa (κw) for ordinal variables and by Cohen's kappa (κ) for nominal ones. Kappa compares an observed and expected accuracy with the evaluation of not only a single question, but also questions among themselves.

The suggestions of Muñoz and Bangdiwala were used for interpretation of the kappa statistic: < 0 = poor agreement, 0 to 0.20 = fair agreement, 0.20 to 0.45 = moderate agreement, 0.45 to 0.75 = substantial agreement, 0.75 to 1.00 = almost perfect agreement [17, 18].

Inclusion and exclusion criteria for data collection see in Table 2.

**Table 2:** Inclusion and exclusion criteria for data collection

	Inclusion criteria	Exclusion criteria
Face validity	FDs with 5 or more years of practice	FDs with less than 5 years practical experience
Content validity	Practicing GPs, teachers of Family Medicine	Experts who did not practice as FDs or did not have teaching experience
Reliability	Family Doctors	Other specialists

## Results

### Translation

After comparison and discussion of both versions of the questionnaire at an expert panel meeting some grammatical issues were cleared and the final version of the Ukrainian questionnaire was accepted.

### Face validity

The face validity was carried out by the participants who mentioned that the contents of the questionnaire were straightforward, comprehensible, and was appropriate for their intended use. Impact scores for all questions were greater than 1.5.

### Content validity

In our study CVI > 0.79 and CVR > 0.59 were acceptable. See Table 3.

**Table 3:** Content Validity Ratio and Content Validity Indexes of questionnaire

Questions	CVR	CVI for relevance	CVI for clarity
1. How useful do you think the following screening examinations are?	0.82	1	0.82
2. Whom do you consider to be responsible for recommending the following screening examinations?	0.82	1	1
3. Whom do you consider to be responsible for conducting the following screening examinations?	1	1	1
4. To what extent do you follow legal recommendations on screening examinations in your practice ?	0.82	1	1
5. What further recommendations do you make as a part of routine practice procedure?	0.27	0.62	0.60
6. How often do you conduct the following screening examinations in your practice yourself?	0.64	1	1

Comment: The list of different screening investigations (presented in the Additional file 1: Appendix) was offered for each of the 6 questions

As Table 1 shows, question 5 didn’t have an acceptable CVR and SVI indexes that is why this question was removed from our questionnaire.

### Reliability testing

The internal consistency was evaluated by using Cronbach’s alpha method. To be acceptable, the internal consistency had to be above 0.7. The questionnaire’s internal consistency was 0.85.

Test–retest reliability was analyzed by weighted kappa (κw) for ordinal variables (Questions 1,4,5) and by Cohen's kappa (κ) for nominal ones(Questions 2,3).

According to the results of weighted kappa and Cohen's kappa coefficients, test–retest agreement of the questionnaire was moderate for 29%, substantial for 38%, and almost perfect for 5% of the items (*p* ≤ 0.05). Kappa coefficients were not computed for 10% of items as there was no variability in the assessments. In 15% of items, test–retest agreement was not significant (*p* > 0.05).

The distribution of weighted kappa (Questions 1,4,5) and Cohen's kappa (Questions 2,3) coefficients for the single items are shown in Tables 4, 5, 6, 7 and 8.

**Table 4:** Weighted kappa coefficients of the items for the question 1of the questionnaire

Question 1. How useful do you think the following screening examinations are?				
	**Weighted Kappa (κw)**	***p*-value**	**Lower 95% Asymptotic CI Bound**	**Upper 95% Asymptotic CI Bound**
**Colonoscopy**^a^	0.308	0.011	0.057	0.559
**FOBT**^a^	0.421	0.000	0.146	0.696
**PSA**^a^	0.529	0.000	0.282	0.777
**Skin cancer screening**^a^	0.389	0.003	0.114	0.664
**Rectum palpation**^a^	0.318	0.017	0.054	0.582
**Mammography**^a^	0.300	0.029	0.048	0.553
**Breast palpation**^a^	0.432	0.001	0.165	0.700
**PAP test**^a^	0.103	0.401	-0.133	0.340
**Blood pressure measurement**^b^	0.392	0.032	0.073	0.712
**BMI assessment**^b^	0.560	0.000	0.305	0.814
**Lipid profile assessment**^b^	0.438	0.002	0.196	0.679
**Fasting plasma glucose, OGTT, HbA1c assessment**^b^	0.714	0.000	0.445	0.983
**HbsAg/antiHCV test**^b^	0.576	0.000	0.357	0.795
**HIV test**^b^	0.534	0.000	0.311	0.757
**PHQ questionnaire**^b^	0.405	0.002	0.166	645
**CBC test**^c^	0.583	0.000	0.355	0.811
**Urine test**^c^	0.588	0.000	0.336	0.840
**ECG**^c^	0.474	0.001	0.148	799
**Lungs X Ray**^c^	0.433	0.002	0.078	0.788

^a^ Methods of examinations taken from the original prototype of the German questionnaire

^b^ Methods of the examination with high level of evidence (A,B) recommended by European guidelines as a part of screening

^c^ Methods of investigations which are still used in Ukraine as a part of old-fashioned annual routine check ups

**Table 5:** Cohen's kappa coefficients for the items from the question 2 of the questionnaire

Question 2. Whom do you consider to be responsible for recommending the following screening examinations?		
	**Cohen's Kappa (κ)**	***p*-value**
**Colonoscopy**^a^	0.714	0.000
**FOBT**^a^	0.829	0.000
**Skin cancer screening**^a^	0.832	0.000
**PSA test**^a^	0.829	0.000
**Rectum palpation**^a^	0.515	0.004
**Mammography**^a^	0.471	0.019
**Breast palpation**^a^	0.176	0.384
**Lipid profile assessment**^b^	0.495	0.015
**Fasting plasma glucose, OGTT, HbA1c assessment**^b^	0.647	0.001
**HbsAg/antiHCV test**^b^	0.442	0.028
**HIV test**^b^	0.333	0.098
**PHQ questionnaire**^b^	0.391	0.054
**Lungs X Ray**^c^	0.238	0.243

Items with absence of variability were not reported in Table 4

^a^ Methods of examinations taken from the original prototype of the German questionnaire

^b^ Methods of the examination with high level of evidence (A,B) recommended by European guidelines as a part of screening

^c^ Methods of investigations which are still used in Ukraine as a part of old-fashioned annual routine check ups

**Table 6:** Cohen's kappa coefficients of the items from the question 3 of the questionnaire

Question 3. Whom do you consider to be responsible for conducting the following screening examinations?		
	**Cohen's Kappa (k)**	***p* Value**
**Colonoscopy**^a^	-0.071	0.648
**FOBT**^a^	0.664	0.001
**Skin cancer screening**^a^	0.583	0.003
**PSA test**^a^	0.385	0.017
**Rectum palpation**^a^	0.467	0.007
**Mammography**^a^	0.231	0.077
**Breast palpation**^a^	0.273	0.127
**PAP test**^a^	0.415	0.027
**Lipid profile**^b^	0.628	0.002
**Fasting plasma glucose,OGTT,HbA1**^b^	0.467	0.007
**HbsAg/antiHCV test**^b^	0.583	0.003
**HIV test**^b^	0.486	0.017
**PHQ questionnaire**^b^	0.489	0.015
**Lungs X Ray**^c^	0.647	0.001

Items with absence of variability were not reported in the Table 5

^a^ Methods of examinations taken from the original prototype of the German questionnaire

^b^ Methods of the examination with high level of evidence (A,B) recommended by European guidelines as a part of screening

^c^ Methods of investigations which are still used in Ukraine as a part of old-fashioned annual routine check ups

**Table 7:** Weighted kappa coefficients of the items for the question 4 of the questionnaire

Question 4. To what extent do you follow legal recommendations on screening examinations in your practice?				
	**Weighted Kappa (κw)**	***p*-value**	**Lower 95% Asymptotic CI Bound**	**Upper 95% Asymptotic CI Bound**
**Colonoscopy**^a^	0.634	0.000	0.331	0.936
**FOBT**^a^	0.494	0.001	0.188	0.800
**Skin cancer screening**^a^	0.622	0.000	0.323	0.920
**PSA test**^a^	0.439	0.004	0.101	0.776
**Rectum palpation**^a^	0.625	0.002	0.293	0.957
**Mammography**^a^	0.357	0.027	0.012	0.702
**Breast palpation**^a^	0.464	0.006	0.085	0.843
**PAP test**^a^	0.347	0.011	0.038	0.655
**Blood pressure measurement**^b^	0.789	0.000	0.334	1.245
**BMI assessment**^b^	0.486	0.004	0.094	0.878
**Lipid profile assessment**^b^	0.298	0.080	-0.091	0.688
**Fasting plasma glucose, OGTT, HbA1c assessment**^b^	0.724	0.000	0.323	1.125
**HbsAg/antiHCV test**^b^	0.463	0.002	0.144	0.781
**HIV test**^b^	0.409	0.005	0.103	0.715
**PHQ questionnaire**^b^	0.455	0.002	0.182	0.727
**CBC test**^c^	0.547	0.001	0.054	1.040
**Urine test**^c^	0.547	0.001	0.054	1.040
**ECG**^c^	0.621	0.000	0.166	1.076
**Lungs X Ray**^c^	0.535	0.002	0.101	0.969

^a^ Methods of examinations taken from the original prototype of the German questionnaire

^b^ Methods of the examination with high level of evidence (A,B) recommended by European guidelines as a part of screening

^c^ Methods of investigations which are still used in Ukraine as a part of old-fashioned annual routine check ups

**Table 8:** Weighted kappa coefficients of the items for the question 5 of the questionnaire

Question 5. What of the following recommendations do you perform in your routine practice?				
	**Weighted Kappa (κw)**	***p*-value**	**Lower 95% Asymptotic CI Bound**	**Upper 95% Asymptotic CI Bound**
**Colonoscopy**^a^	0.298	0.043	0.008	0.588
**FOBT**^a^	0.435	0.003	0.103	0.768
**Skin cancer screening**^a^	0.386	0.005	0.099	0.673
**PSA test**^a^	0.327	0.011	0.066	0.587
**Rectum palpation**^a^	0.005	0.073	-0.291	0.302
**Mammography**^a^	0.111	0.463	-0.212	0.434
**Breast palpation **^a^	0.768	0.000	0.519	1.017
**PAP test**^a^	0.176	0.238	-0.148	0.501
**Blood pressure measurement**^b^	0.396	0.023	-0.057	0.849
**BMI assessment**^b^	0.520	0.001	0.217	0.823
**Lipid profile assessment**^b^	0.263	0.072	-0.043	0.569
**Fasting plasma glucose, OGTT, HbA1c assessment**^b^	0.486	0.002	0.158	0.814
**HbsAg/antiHCV test**^b^	0.400	0.009	0.102	0.698
**HIV test**^b^	0.355	0.015	0.043	0.667
**PHQ questionnaire**^b^	0.400	0.008	0.084	0.716
**CBC test**^c^	0.380	0.023	-0.001	0.762
**Urine test**^c^	0.429	0.011	0.050	0.807
**ECG**^c^	0.312	0.067	-0.059	0.684
**Lungs X Ray**^c^	0.378	0.020	-0.002	0.758

^a^ Methods of examinations taken from the original prototype of the German questionnaire

^b^ Methods of the examination with high level of evidence (A,B) recommended by European guidelines as a part of screening

^c^ Methods of investigations which are still used in Ukraine as a part of old-fashioned annual routine check ups

## Discussion

This study aimed to translate the original German prototype of “Readiness of GPs to recommend and implement evidence-based screening recommendations questionnaire”, and to test it concerning validity and reliability of the Ukrainian version was modified by including questions about evidence based screening of cardiovascular risks, infectious diseases, type 2 diabetes mellitus, depression, and some old-fashioned annual checkups which are still commonly used in routine family doctors’ practice (i.e. CBC, urine analysis, lungs X-ray).

First of all we started from forward–backward translation of the original English version of the questionnaire into Ukrainian language. Face validation process confirmed that the questions of the questionnaire were adequately structured, grammatically correct, and understandable. During the quantitative part of content validation process CVRs and CVIs indexes were calculated for all the questions. As a result only question number 5 from the questionnaire was not essential, relevant, and clear enough (CVR and CVIs were not acceptable). The GPs are not yet directly concerned by this question. That is why it was removed from the questionnaire.

There is no a state supported evidence-based screening program in Ukraine yet and some of GPs still are used to use post-Soviet routine investigations as a part of routine annual checkups (i.e. CBC, urine test) [9]. That is why we added those investigations to the general list of investigations (See Additional file 1: Appendix).

Internal consistency of the items with the sample size of 57, assessed by using Cronbach’s alpha method was acceptable.

For the assessment of stability of the questionnaire, test–retest reliability was performed, where 19 GPs filled in the same questionnaire twice over a period of two weeks. According to the results of statistical analysis (Cohen's kappa and weighted kappa coefficients), test–retest reliability of 84,2% of items was acceptable (moderate for 29,4%, substantial for 39%, almost perfect for 5,3% and not computed for 10,5% of items because of no differences in respondents’' answers for the first and for the second time).

Up to the authors, the rest (15.8%) of items were not significant due to GPs doubts concerning what the right screening is.

The Ukrainian version of the questionnaire is reliable and adapted to the sociodemographic primary care context. The taken out questions did not directly concern the GPs in Ukraine because they don’t perform such procedures in their practice.

Why is this questionnaire useful in the Ukrainian primary care setting? In absence of national evidence-based screening guidelines supported by the state, GPs remain the key medical professionals in promoting and advising evidence-based screening strategies to the community.

We are aware that the current war situation in Ukraine will not foster screening implementation into clinical practice of Ukrainian Family doctors.

Before the new national screening protocol is developed it’s crucial to form the correct way of understanding by primary health professionals the right screening strategy of common non-communicable diseases.

Nowadays, Ukraine bears the second largest HIV epidemic in Eastern Europe and Central Asia. In 2019, an estimated number of 250.777people living has HIV (PLHIV) in Ukraine (Spectrum) and 2.977 AIDS-related deaths (14% less than in 2018) and 16.405 newly diagnosed HIV cases (4% more than in 2018) were reported [19]. That’s why HIV has to be included in the list of mandatory to be screened diseases in primary medical care Ukraine.

According to the WHO 2017 data, Ukraine belongs to the countries with the average spread of Hepatitis C: approximately 3% of the population, i.e. 1.170.000 people, were infected. However, the results of selective monitoring of risk groups showed that the level of infection by Hepatitis C in some of them exceeds the average indicators and reaches 40–60% [20]. That’s why HCV has to be included in the list of mandatory to be screened diseases in Ukraine.

Cervical cancer is a frequent disease in Ukraine. Among all women cancers, it ranks 5th for incidence and 6th for mortality. While first year mortality shows some decline, efforts on earlier detection are mandatory [21]. That’s why cervical cancer has to be included in the list of mandatory to be screened diseases in primary medical care Ukraine.

The cardiovascular disease (CVD) mortality level in Ukraine is 772.1 per 100 000 for men and 440.9 per 100 000 for women. This accounts for 68,0% of the total mortality in the country [22, 23]. That’s why CVD screening has to be included in the list of mandatory to be screened diseases in primary medical care Ukraine.

Thus, this new and reliable questionnaire has a direct practical implication to determine GP’s readiness to get involved in evidence-based screening implementation and to meet the screening need in their country.

### Strengths and limitations

This is the first concrete step to get involved GPs in Ukraine into screening routines. Our sample size of test–retest reliability calculation after the second-time questioning included only 19 doctors which is small. Initially we had a bigger number of participants (*n* = 60) but due to the new COVID-19 pandemic wave at that time which caused family doctors' workload the response rate for the second-time questioning was much lower (*n* = 19).

## Conclusions

The original German prototype of “Readiness of GPs to recommend and implement evidence-based screening recommendations questionnaire”, has been translated to Ukrainian and tested concerning validity and reliability. The Ukrainian version was modified by including questions about screening of cardiovascular, infectious diseases, type 2 diabetes mellitus, depression, and prior local routine screening investigations (CBC, urine analysis, lungs X-ray). It can now be used as a reliable tool to determine GP’s readiness to get involved in evidence-based screening implementation and to meet the screening need in their country.

## Supplementary Information

**Additional file 1. **Readiness of general practitioners to recommend andimplement evidence-based screening recommendations questionnaire.

## Data Availability

Only summarized data sets generated or analyzed during the current study are included in this article.

## Abbreviations

CBC: Complete blood count
CVD: Cardio-vascular diseases
CVI: Content validity index
CVR: Content validity ratio
FD: Family doctor
GP: General practitioner
HbA1c: Glycated hemoglobin
HCV: Hepatitis C virus
HIV: Human immunodeficiency virus
κ: Cohen's kappa
κw: Weighted kappa
OGTT: Oral glucose tolerance test
WONCA: World Organization of National Colleges and Academies of Family Medicine
