# Supplementary material for: Validation of a modified questionnaire to assess Ukrainian Family Physicians’ readiness to implement the evidence-based screening recommendations into their clinical practice, using a mixed method study
Source: BMC Prim Care. 2022 Sep 7;23:225. doi: 10.1186/s12875-022-01818-4 (PMC9449945; doi:10.1186/s12875-022-01818-4)
Supplement: Supplementary file 1 — Additional file 1. Readiness of general practitioners to recommend andimplement evidence-based screening recommendations questionnaire. [file 12875_2022_1818_MOESM1_ESM.docx]

**“Readiness of general practitioners to recommend and implement evidence-based screening recommendations questionnaire”.**

**1.How useful do you think the following screening examinations are?**

Please cross one box per line (per investigation).

|  | **Not useful** | **Of little use** | **Fairly useful** | **Useful** |
| --- | --- | --- | --- | --- |
| Blood pressure measurement |  |  |  |  |
| BMI assessment |  |  |  |  |
| Blood lipid profile assessment |  |  |  |  |
| Fasting plasma glucose,OGTT,HbA1c assessment |  |  |  |  |
| Colonoscopy |  |  |  |  |
| FOBT |  |  |  |  |
| Skin cancer screening |  |  |  |  |
| PSA test |  |  |  |  |
| Digital-rectal examination |  |  |  |  |
| Mammography |  |  |  |  |
| Breast palpation |  |  |  |  |
| Pap test |  |  |  |  |
| HbsAg/Anti-HCV tests |  |  |  |  |
| HIV test |  |  |  |  |
| PHQ questionnaire |  |  |  |  |
| CBC test |  |  |  |  |
| Urine analysis |  |  |  |  |
| ECG |  |  |  |  |
| Lungs X-ray |  |  |  |  |

**2.Whom do you consider to be responsible for recommending the following screening examinations?**

Please cross one box per line.

|  | **GP** | **Specialist** |
| --- | --- | --- |
| BP measurement |  |  |
| BMI assessment |  |  |
| Blood lipid profile assessment |  |  |
| Fasting plasma glucose, OGTT, HbA1c assessment |  |  |
| Colonoscopy |  |  |
| FOBT |  |  |
| Skin cancer screening |  |  |
| PSA test |  |  |
| Digital-rectal examination |  |  |
| Mammography |  |  |
| Breast palpation |  |  |
| Pap test |  |  |
| HbsAg/Anti-HCV |  |  |
| HIV test |  |  |
| PHQ questionnaire |  |  |
| CBC test |  |  |
| Urine analysis |  |  |
| ECG |  |  |
| Lungs X-ray |  |  |

**3.Whom do you consider to be responsible for conducting the following screening examinations?**

Please cross one box per line (per investigation).

|  | **GP** | **Specialist** |
| --- | --- | --- |
| BP measurement |  |  |
| BMI assessment |  |  |
| Blood lipid profile assessment |  |  |
| Fasting plasma glucose,OGTT,HbA1c assessment |  |  |
| Colonoscopy |  |  |
| FOBT |  |  |
| Skin cancer screening |  |  |
| PSA test |  |  |
| Digital-rectal examination |  |  |
| Mammography |  |  |
| Breast palpation |  |  |
| Pap test |  |  |
| HbsAg/Anti-HCV tests |  |  |
| HIV test |  |  |
| PHQ questionnaire |  |  |
| CBC test |  |  |
| Urine analysis |  |  |
| ECG |  |  |
| Lungs X-ray |  |  |

**4.To what extent do you follow legal recommendations on screening examinations in your practice?**

Please cross one box per line (per investigation).

|  | **Making the**  **recommendation is part**  **of routine practice**  **procedure.** | **I/ my staff irregularly**  **make the recommendations to**  **practice**  **patients.** | **I/ my staff never make the**  **recommendations to practice**  **patients or only in special**  **cases.** |
| --- | --- | --- | --- |
| BP measurement |  |  |  |
| BMI assessment |  |  |  |
| Blood lipid profile assessment |  |  |  |
| Fasting plasma glucose,OGTT,  HbA1c assessment |  |  |  |
| Colonoscopy |  |  |  |
| FOBT |  |  |  |
| Skin cancer screening |  |  |  |
| PSA test |  |  |  |
| Digital-rectal examination |  |  |  |
| Mammography |  |  |  |
| Breast palpation |  |  |  |
| Pap test |  |  |  |
| HbsAg/Anti-HCV tests |  |  |  |
| HIV test |  |  |  |
| PHQ questionnaire |  |  |  |
| CBC test |  |  |  |
| Urine analysis |  |  |  |
| ECG |  |  |  |
| Lungs X-ray |  |  |  |

**How often do you conduct the following screening examinations in your practice yourself?**

5.Please cross one box per line (per investigation).

|  | **never** | **irregularly** | **regularly** |
| --- | --- | --- | --- |
| BP measurement |  |  |  |
| BMI assessment |  |  |  |
| Blood lipid profile assessment |  |  |  |
| Fasting plasma glucose,OGTT,HbA1c assessment |  |  |  |
| Colonoscopy |  |  |  |
| FOBT |  |  |  |
| Skin cancer screening |  |  |  |
| PSA test |  |  |  |
| Digital-rectal examination |  |  |  |
| Mammography |  |  |  |
| Breast palpation |  |  |  |
| Pap test |  |  |  |
| HbsAg/Anti-HCV test |  |  |  |
| HIV test |  |  |  |
| PHQ questionnaire |  |  |  |
| CBC test |  |  |  |
| Urine analysis |  |  |  |
| ECG |  |  |  |
| Lungs X-ray |  |  |  |
